# Sex‐specific associations between gut microbiota and skeletal muscle mass in a population‐based study

**DOI:** 10.1002/jcsm.13096

**Published:** 2022-10-11

**Authors:** Chul‐Hyun Park, Eun‐Ju Lee, Hyung‐Lae Kim, Yong‐Taek Lee, Kyung Jae Yoon, Han‐Na Kim

**Affiliations:** ^1^ Department of Physical and Rehabilitation Medicine, Kangbuk Samsung Hospital Sungkyunkwan University School of Medicine Seoul Republic of Korea; ^2^ Medical Research Institute, Kangbuk Samsung Hospital Sungkyunkwan University School of Medicine Seoul Republic of Korea; ^3^ Department of Biochemistry, College of Medicine Ewha Womans University Seoul Republic of Korea; ^4^ Department of Clinical Research Design and Evaluation, SAIHST Sungkyunkwan University Seoul Republic of Korea; ^5^ Biomedical Institute for Convergence at SKKU Sungkyunkwan University School of Medicine Suwon Republic of Korea

**Keywords:** gut microbiome, microbiota, sarcopenia, skeletal muscle mass

## Abstract

**Background:**

A gut–muscle axis through which the microbiome influences skeletal muscle has been hypothesized. However, sex‐specific association between the characteristics of gut microbiota and skeletal muscle mass has not yet been reported. Herein, we performed sex‐specific analyses of faecal microbiota composition for the skeletal muscle mass in a population‐based cohort.

**Methods:**

We collected faecal samples of 1052 middle‐aged participants (621 men and 431 women) who attended health screenings, and we analysed the intestinal microbiota using 16S rRNA gene sequencing. Relative muscle mass was calculated using a bioelectrical impedance analysis and presented as the skeletal muscle mass index [SMI (%) = total appendicular muscle mass (kg)/weight (kg) × 100]. We categorized the subjects into four groups by the quartile of the SMI. Association tests between gut microbiota and SMI were conducted according to the microbial diversity, taxonomic profiling and functional inference in a sex‐stratified manner.

**Results:**

The mean age and SMI of the total participants were 44.8 years (standard deviation [SD], 8.2) and 41.4% (SD, 3.9), respectively. After adjustments for possible covariates such as age, body mass index and regular physical activity, the highest quartile (Q4) group of SMI had higher alpha diversity than the lowest quartile (Q1) group in male participants (coefficient = 10.79, *P* < 0.05, linear regression model), whereas there was no difference in diversity among SMI groups in females. At the species level, *Haemophilus parainfluenzae* (coefficient = 1.910) and *Roseburia faecis* (coefficient = 1.536) were more abundant in the highest SMI (Q4) group than in the lowest SMI (Q1) group in males. However, no significant taxon was observed along the SMI groups in females. The gut microbiota of the lowest SMI group (Q1) was enriched with genes involved in biosynthesis of amino acids and energy generation compared with that of the highest SMI group (Q4) in both sexes, although the significance of the inferred pathways was weak (*P* < 0.05 but the false discovery rate *q* > 0.05).

**Conclusions:**

In this large sample of middle‐aged individuals, this study highlights fundamental sex‐specific differences in the microbial diversity, composition and metabolic pathways inferred from gut microbiota according to SMI. The gut microbiota may provide novel insights into the potential mechanisms underlying the sex dependence of skeletal muscle mass.

## Introduction

Skeletal muscle is a key organ for activities of daily living. The decrease in skeletal muscle mass can cause muscle weakness, physical frailty and functional disability. Age‐related loss of skeletal muscle has been shown to be associated with an increased risk of morbidity (chronic diseases) and all‐cause mortality in previous studies.^1^, ^2^ The loss of muscle mass occurs incipiently from the middle age (~1%/year), and in severe instances, it can lead to a loss of ~50% by the eighth to ninth decade of life.^3^

Gut microbiota particularly influences host health. Skeletal muscle also takes part in the interorgan crosstalk regulating substrate metabolism, immunity and health. Recent studies have reported that gut microbiota has interactions with skeletal muscle mass, known as the gut–muscle axis.^4^, ^5^ Perturbations in the gut microbiota can induce substantial modifications in skeletal muscle physiology and function.^6^ Germ‐free mice lacking a gut microbiota exhibit decreased skeletal muscle mass, strength and mitochondrial function.^7^ Short‐chain fatty acids (SCFAs) produced from the fermentation of indigestible carbohydrates via the gut microbiome may influence the metabolism of skeletal muscle.^6^ The imbalance of microbial diversity induces low‐grade systemic inflammation through altered gut permeability and microbial products, which could reduce the skeletal muscle mass. This relationship between the gut microbiota and skeletal muscle has also been demonstrated in studies using probiotic and prebiotic interventions.^6^

However, there was rare direct evidence of an association between gut microbiota composition and muscle mass in humans as most previous studies have been performed on animals. To date, few studies have explored the gut–muscle axis in humans. Previous studies investigating the relationship between muscle mass and gut microbiota have focused mainly on the older adults and patients with sarcopenia,^5^, ^8^ although decreased muscle mass has been shown to be related to various health outcomes, not only in older adults but also among middle‐aged populations.^9^ Recently, a few studies on the association between gut microbiota and muscle parameters have been reported.^10^, ^11^, ^12^ To the best of our knowledge, however, no large population‐based study, including young‐ and middle‐aged individuals, has investigated the association of gut microbiota with skeletal muscle mass. In addition, various previous studies have shown a strong association between sex and loss of muscle mass on human health,^9^ and sex specificity is known to influence the composition of the gut microbiome.^13^

In this study, we hypothesized that the gut microbiome was associated with the skeletal muscle mass. As the muscle mass differs between men and women, we further hypothesized that there were sex‐specific microbiome signatures associated with the muscle mass that have not been investigated to date. Here, we investigated the gut microbiome compositions according to the amount of muscle mass in the large population‐based cohort and examined whether there are sex‐based disparities in the association between skeletal muscle mass and gut microbiota.

## Methods

### Study subjects

**Figure 1:**
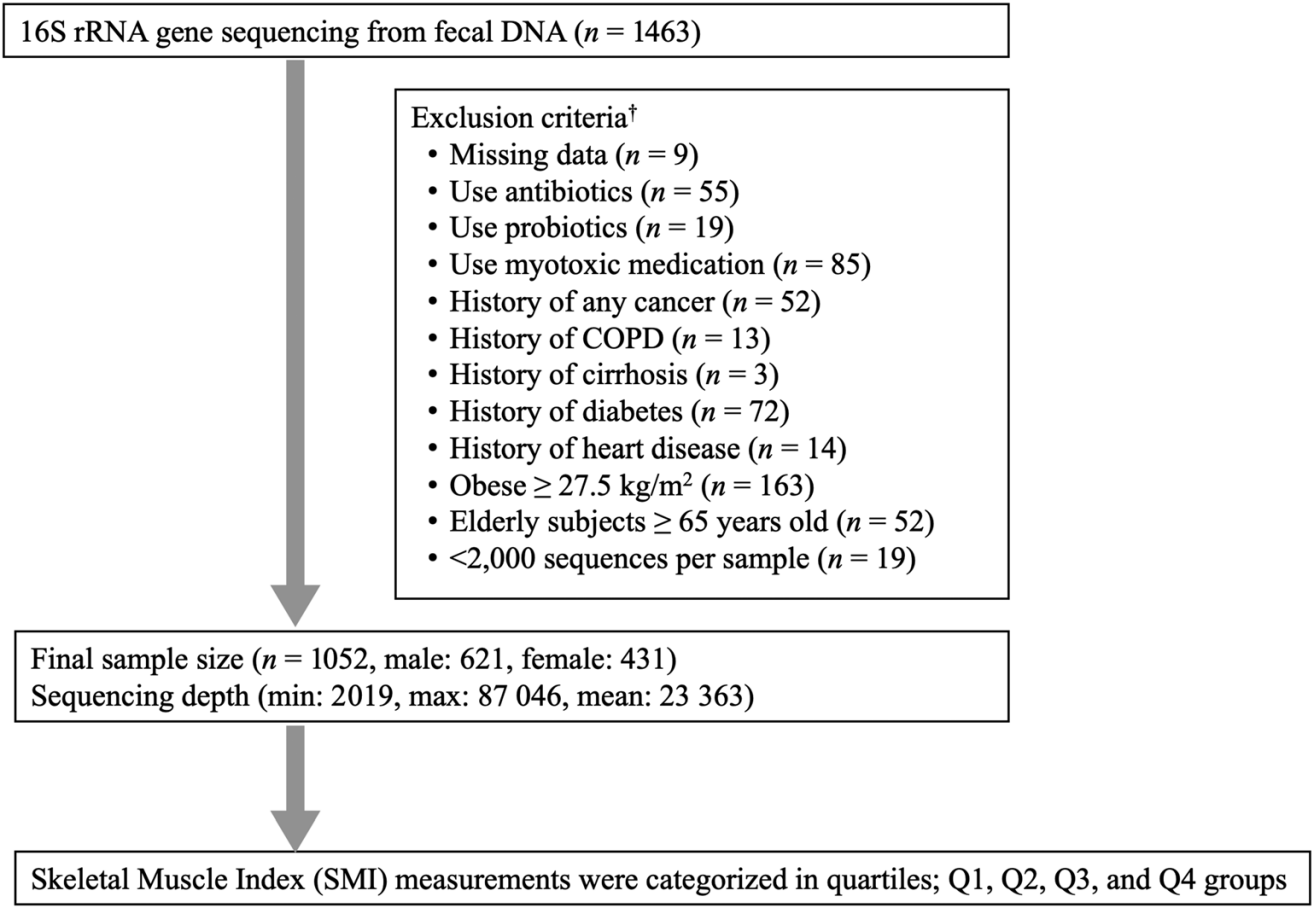
Enrolment of study subjects. ^†^Some individuals met several exclusion criteria. COPD, chronic obstructive pulmonary disease

We recruited 1463 Korean men and women aged 25 to 78 years who underwent a comprehensive annual or biennial physical examination between June 2014 and September 2014 at Kangbuk Samsung Hospital Healthcare Screening Center in the Republic of Korea. Faecal samples were collected from participants aged from 25 to 64 years. Participants were excluded according to the exclusion criteria described below (*Figure* *1*). We excluded 164 subjects as per the following criteria: missing data (*n* = 9), use of antibiotics within 6 weeks before enrolment (*n* = 55), use of probiotics within 4 weeks before enrolment (*n* = 19), use of myotoxic medication (*n* = 85), history of any cancer (*n* = 52), history of chronic obstructive pulmonary disease (*n* = 13), history of cirrhosis (*n* = 3), history of diabetes (*n* = 72), history of heart disease (*n* = 14), obese ≥27.5 kg/m^2^ (*n* = 163) based on World Health Organization (WHO) Asia body mass index (BMI) cut‐off,^14^ elderly subjects ≥65 years old (*n* = 52) and samples with less than 2000 sequences (*n* = 19). None of the included patients had tuberculosis. Some individuals met more than one exclusion criterion, and a total of 1052 participants were included in the final analysis.

This study protocol was conducted according to the Declaration of Helsinki and was approved by the Institutional Review Board (IRB) of Kangbuk Samsung Hospital (IRB No. KBSMC 2021‐12‐043). Written informed consent was obtained from all participants after the nature, and possible consequences of the study were explained.

### Data collection and group definitions

Subjects completed self‐administered questionnaires (e.g., medical history review). Dietary consumption was assessed using a 103‐item self‐administered food frequency questionnaire (FFQ) developed for use in Korea.^15^ This validated FFQ was designed to measure usual food consumption during the previous year. Blood samples were collected by a trained nurse in the morning from the antecubital vein of patients who had fasted for more than 12 h. Serum biochemical parameters included fasting insulin, glucose, triglycerides and alanine aminotransferase (ALT). Physical activity was assessed using the International Physical Activity Questionnaire‐116 Short Form. Participants who performed vigorous exercise ≥3 times a week for over 20 min per session were categorized as a regular physical activity group.^16^ Anthropometric measurements, including height, weight, skeletal muscle mass and fat mass, were assessed. The skeletal muscle mass (kg) was calculated using a bioelectric impedance analyser (BIA) of eight‐point tactile electrodes (InBody 720, Biospace Co., Seoul, Korea). The BIA was calibrated and validated for accuracy and reproducibility of body composition index.^17^ For adjustment for the effect of body weight, the skeletal muscle mass index (SMI) was calculated with the following formula: SMI (%) = skeletal muscle mass (kg)/weight (kg) × 100, based on the previously established method.^18^ Subjects were categorized into four groups according to SMI quartiles: Q1 (first quartile), Q2 (second quartile), Q3 (third quartile) and Q4 (fourth quartile).

### DNA extraction from faecal samples and 16S rRNA gene sequencing

Faecal samples were immediately frozen at −20°C after collection and stored at −70°C within 24 h. DNA extraction from faecal samples was performed within 1 month of storage using the MOBio PowerSoil® DNA Isolation Kit (MO BIO Laboratories, Carlsbad, CA, USA) using the bead beating step according to the manufacturer's instructions. Variable regions V3 and V4 of the 16S rRNA gene were amplified by polymerase chain reaction (PCR) with universal primers. Libraries were pooled for sequencing using the full complement of Nextera XT indices and paired‐end sequenced on the Illumina MiSeq platform (Illumina, San Diego, CA, USA) according to the manufacturer's instructions.

The Divisive Amplicon Denoising Algorithm, Version 2 (DADA2) plugin of the Quantitative Insights into Microbial Ecology (QIIME2) package (Version 2019.7, https://qiime2.org) was used to perform sequence quality control and construct a feature table of amplicon sequence variants (ASVs) that is regarded as 100% operational taxonomic units (OTUs). We applied the contingency‐based filtering to remove features that are present in only a single sample in our feature table. Taxonomy was assigned to all ASVs by searching against the National Center for Biotechnology Information (NCBI) Nucleotide and Taxonomy databases (NCBI‐RefSeq, accessed on 9 June 2021) using RESCRIPt within QIIME2. In the taxonomic assignment of ASVs, the average of the confidence is 0.948 (standard deviation [SD], 0.08).

### Statistical analysis

Previous data show that skeletal muscle mass differs according to sex^19^; therefore, we compared the SMI between males and females. The SMIs were compared using Student's *t* tests. As the SMI differed by sex, association analyses between SMI and gut microbiota were conducted separately for males and females. To find out a linear trend of baseline characteristics according to increasing SMI quartiles, one‐way analysis of variance (ANOVA) for continuous variables and *χ* ^2^ tests for categorical variables were performed. The right‐skewed variables (insulin, glucose and triglycerides) were log transformed, and a one‐way ANOVA was performed. We controlled for age, BMI and regular physical activity to determine if there was a significant association between gut microbiota and skeletal muscle mass.

For diversity analysis, the feature table was rarefied to 2019 sequences per sample by random subsampling in the QIIME2 package (Version 2021.11, https://qiime2.org). To evaluate alpha diversity, we computed the number of ASVs observed in each sample, Faith's phylogenetic diversity (Faith's PD; measures of biodiversity that incorporate phylogenetic differences between species), Shannon's index (calculates richness and diversity using a natural logarithm) accounting for both evenness and richness and Pielou's evenness (measures of relative evenness of species richness). The estimated marginal means (EMMs) analysis was used to show the adjusted mean of each SMI quartile group, controlling for covariates such as age, BMI and regular physical activity. For the linear regression models, Q1 was set as the reference group and compared with the Q2, Q3 and Q4 groups, respectively. To estimate the dissimilarity between samples, beta diversity was calculated using the UniFrac distance to estimate dissimilarity among group members by incorporating the phylogenetic distances between ASVs. Unweighted and weighted UniFrac distances were calculated to determine the presence/absence and abundance of ASVs, respectively. Non‐phylogenetic beta‐diversity indices such as Bray–Curtis dissimilarities were also used for abundance data. Pairwise permutational multivariate ANOVA (PERMANOVA) with 999 random permutations using *adonis* function was performed to test the significance of differences between groups. Age, BMI and regular physical activity were included in the formula of *adonis* as covariates. Basic statistical analyses were performed using RStudio (Version 1.3.1073, Boston, MA, USA), and plots of microbial diversity were depicted using the ggplot2 package (Version 3.3.2) in RStudio.

To robustly investigate the significant differences in the relative taxa abundances from the phylum to species levels among groups, we used two statistical tools from R (Version 4.0.2): analysis of composition of microbiomes (ANCOM)‐II (https://github.com/FrederickHuangLin/ANCOM, accessed on 17 May 2022) and generalized linear models implemented in multivariate association with linear models (MaAsLin2). After adjusting for age, BMI and regular physical activity, we compared the abundance of taxa between the Q1 and Q4 groups in a pairwise manner based on microbial diversity results. ANCOM compares the relative abundance of taxa among groups by the log ratio of the abundance of each taxon to that of the remaining taxa, one at a time. The final significance was expressed in the empirical distribution of *W* at each taxonomic level. We used the taxa‐wise false discovery rate (FDR) option with the significance level set to FDR < 0.05 to generate *W* statistics and a threshold of 0.6 for declaring a significant association. For the MaAsLin2 models, Q1 was set as the reference group and compared with Q4. After adjusting for covariates such as age, BMI and regular physical activity, we estimated the exponentiated coefficients by comparing the highest quartile (Q4) of SMI to the lowest quartile (Q1).

To predict metagenome functional content from 16S rRNA gene surveys, we predicted the functional pathways from the MetaCyc metabolic pathway database using PICRUSt2 (v2.4.2, accessed on 22 April 2020). The predicted functional pathways were compared among the groups using statistical analysis of taxonomic and functional profiles (STAMP) Version 2.1.3. Statistical differences in the pathways were tested using Welch's *t* test with a Benjamini–Hochberg FDR correction (*q* value <0.05) to adjust for multiple testing.

References to the ‘DNA extraction from fecal samples and 16S rRNA gene sequencing’ and ‘Statistical analysis’ are cited in the Methods and References in the supporting information.

## Results

### Demographics of subjects

This study included 1052 participants, consisting of 621 males (*Table* *1*) and 431 females (*Table* *2*). The mean age and SMI of the total subjects were 44.8 years (SD, 8.2) and 41.4% (SD, 3.9), respectively. There was a significant difference in the mean age between males and females (male, mean 45.4 years [SD, 8.2]; female, mean 44.1 years [SD, 8.1]) (*P* = 0.015). The mean (SD) of SMI was significantly higher in males (45.5 [8.2]) than in females (44.1 [8.1]) (*P* < 0.001).

**Table 1:** Baseline characteristics based on quartiles of skeletal muscle mass index in males

Characteristics	Overall	Quartiles (Q) of skeletal muscle mass index				*P* for trend
		Q1	Q2	Q3	Q4	
Subjects (*n*)	621	155	155	155	156	
Age (years)^a^	45.4 (8.2)	46.3 (8.6)	46.5 (7.9)	44.5 (7.3)	44.2 (8.7)	0.004
BMI (kg/m^2^)^a^	23.8 (2.1)	24.9 (1.5)	24.3 (1.8)	23.6 (1.9)	22.4 (2.0)	<0.001
Skeletal muscle mass (kg)^a^	31.0 (3.3)	29.3 (2.7)	30.9 (3.1)	31.8 (3.6)	31.8 (3.3)	<0.001
Skeletal muscle mass index (%)^a^	43.8 (2.6)	40.4 (1.4)	43.0 (0.5)	44.7 (0.5)	46.9 (1.2)	<0.001
Fat mass (kg)^a^	15.7 (3.9)	20.0 (2.7)	16.8 (2.1)	14.7 (2.2)	11.4 (2.3)	<0.001
Regular physical activity (%)	15.3	12.0	7.3	13.2	28.4	<0.001
Hypertension (%)	11.8	13.5	16.8	9.7	7.1	0.042
Insulin (uIU/mL)^b^	5 (3–7)	6 (4–8)	5 (3–7)	5 (4–6)	4 (3–5)	<0.001
Glucose (mg/dL)^b^	94 (89–101)	93 (87–99)	95 (89–101)	94 (89–99)	94 (90–98)	0.721
Triglycerides (mg/dL)^b^	111 (81–156)	132 (92–171)	125 (83–167)	106 (67–145)	84 (61–107)	<0.001
ALT (U/L)^b^	23.4 (15.4)	25.4 (15.5)	24.5 (14.7)	24.0 (17.5)	19.6 (13.2)	0.001
Subjects with nutrient information (*n*)	462	111	118	121	112	
Total calorie (kcal/day)^a^	1468.0 (621.6)	1533.8 (588.3)	1351.1 (580.9)	1382.3 (646.2)	1618.6 (642.7)	0.271
Protein (g/day)^a^	50.4 (25.0)	53.9 (25.1)	45.7 (21.8)	47.2 (25.3)	55.1 (27.7)	0.621
Fat (g/day)^a^	29.2 (19.3)	31.8 (19.7)	25.3 (16.4)	27.8 (18.7)	32.3 (21.9)	0.63
Carbohydrate (g/day)^a^	246.5 (105.6)	253.6 (99.3)	230.7 (106.6)	232.1 (102.5)	271.9 (105.4)	0.198
Fibre (g/day)^a^	3.6 (2.1)	3.8 (2.1)	3.4 (1.9)	3.6 (2.2)	3.8 (2.1)	0.430

*Note*: One‐way ANOVA for continuous variables and *χ* ^2^ tests for categorical variables were used to compare the baseline characteristics of the participants, and the right‐skewed variables (insulin, glucose and triglycerides) were log transformed for one‐way ANOVA.

Abbreviations: ALT, alanine aminotransferase; ANOVA, analysis of variance; BMI, body mass index; SD, standard deviation.

^a^ Data are expressed as mean (SD).

^b^ Data are expressed as median (interquartile range).

**Table 2:** Baseline characteristics based on quartiles of skeletal muscle mass index in females

Characteristics	Overall	Quartiles (Q) of skeletal muscle mass index				*P* for trend
		Q1	Q2	Q3	Q4	
Subjects (*n*)	431	107	108	108	108	
Age (years)^a^	44.1 (8.1)	46.1 (9.2)	44.9 (7.7)	44.1 (7.1)	41.4 (7.4)	<0.001
BMI (kg/m^2^)^a^	21.6 (2.4)	23.6 (2.1)	22.1 (2.0)	21.3 (1.9)	19.6 (1.7)	<0.001
Skeletal muscle mass (kg)^a^	21.0 (2.4)	20.2 (2.2)	20.7 (2.2)	21.6 (2.6)	21.7 (2.3)	<0.001
Skeletal muscle mass index (%)^a^	38.1 (3.0)	34.3 (1.2)	36.9 (0.5)	39.1 (0.7)	42.0 (1.5)	<0.001
Fat mass (kg)^a^	16.4 (4.4)	21.2 (3.2)	17.7 (2.4)	15.3 (2.3)	11.5 (2.5)	<0.001
Regular physical activity (%)	15.6	15.5	11.3	16.2	19.2	<0.001
Hypertension (%)	5.3	12.1	0	5.6	3.7	0.001
Insulin (uIU/mL)^b^	4 (3–5)	5 (4–6)	5 (4–6)	3 (2–4)	3 (2–4)	<0.001
Glucose (mg/dL)^b^	90 (86–94)	92 (87–97)	90 (87–93)	88 (83–93)	88 (84–92)	<0.001
Triglycerides (mg/dL)^b^	80 (60–110)	97 (71–123)	85 (54–115)	76.0 (52–99)	66 (49–83)	<0.001
ALT (U/L)^b^	15.1 (9.6)	16.7 (11.1)	15.1 (7.3)	15.5 (11.9)	13.3 (6.7)	0.019
Subjects with nutrient information (*n*)	316	79	76	81	80	
Total calorie (kcal/day)^a^	1301.8 (585.2)	1373.1 (626.2)	1289.7 (589.8)	1309.8 (537.9)	1232.7 (587.3)	0.158
Protein (g/day)^a^	44.6 (22.1)	46.8 (23.7)	45.5 (23.1)	44.6 (21.8)	41.6 (19.6)	0.131
Fat (g/day)^a^	26.1 (17.1)	16.9 (18.2)	26.2 (17.9)	25.9 (17.3)	25.4 (15.2)	0.609
Carbohydrate (g/day)^a^	219.9 (103.1)	233.6 (112.9)	216.0 (101.9)	222.2 (91.2)	207.2 (105.6)	0.137
Fibre (g/day)^a^	3.9 (2.5)	4.2 (2.7)	4.0 (2.5)	3.9 (2.4)	3.4 (2.3)	0.052

*Note*: One‐way ANOVA for continuous variables and *χ* ^2^ tests for categorical variables were used to compare the baseline characteristics of the participants, and the right‐skewed variables (insulin, glucose and triglycerides) were log transformed for one‐way ANOVA.

Abbreviations: ALT, alanine aminotransferase; ANOVA, analysis of variance; BMI, body mass index; SD, standard deviation.

^a^ Data are expressed as mean (SD).

^b^ Data are expressed as median (interquartile range).

In both males and females, there was no significant trend of nutritional information such as total calorie, protein, fat, carbohydrate and fibre according to SMI quartiles. In males, the variables with downtrend relative to increasing SMI quartiles were age, BMI, fat mass, hypertension, insulin, triglycerides and ALT. The variables with uptrend relative to SMI quartiles were skeletal muscle mass, SMI and regular physical activity. In females, the variables with downtrend relative to increasing SMI quartiles were age, fat mass, hypertension, insulin, glucose, triglycerides and ALT. The variables with uptrend relative to SMI quartiles were skeletal muscle mass, SMI and regular physical activity in males. The variables with uptrend relative to SMI quartiles were skeletal muscle mass and SMI.

### Alpha and beta diversity among quartile groups of skeletal muscle mass index

**Figure 2:**
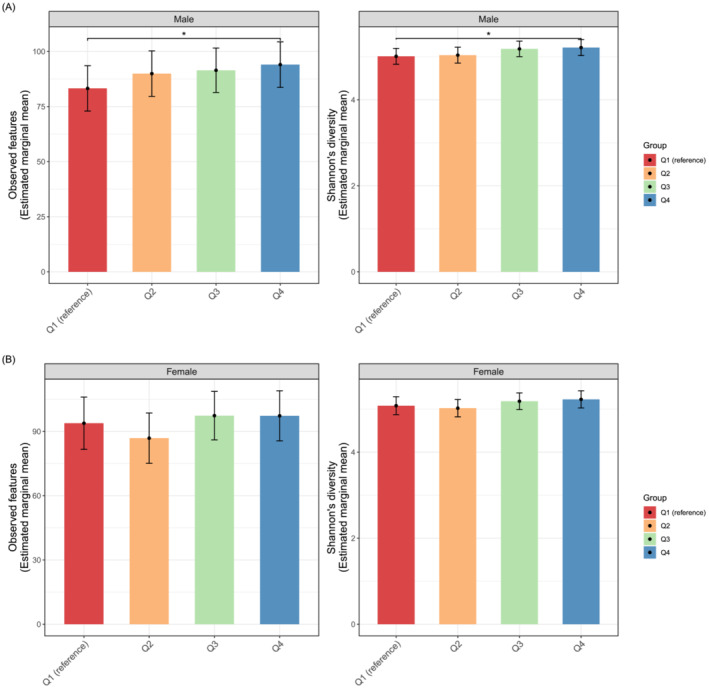
Alpha diversity of the gut microbiota among the skeletal muscle mass index groups in (A) males and (B) females. Statistics were calculated using a linear regression model adjusted for age, body mass index and regular physical activity as covariates. The Q1 group was set as the reference group. *P < 0.05. The colour bars represented estimated marginal means values adjusted for the covariates, and high–low bars represent 95% confidence intervals.

In the total participants, the sequencing depth ranged from 2019 to 87 046 reads per sample (mean = 23 363), and the number of features was 3689 in 1052 subjects after contingency‐based filtering of features. We found no significant imbalance of read depths across the SMI quartile groups in males (*P* = 0.452, ANOVA) and females (*P* = 0.718, ANOVA) (*Figure* *S1*), although the read depths were variable across total samples. To better identify the relationship between skeletal muscle mass and gut microbial diversity, we controlled for covariates such as age, BMI and regular physical activity. In males, we found that the highest quartile (Q4) group of SMI had high alpha diversity than the lowest quartile (Q1) group in males across SMI quartile groups: observed features (coefficient = 10.79, *P* < 0.05, linear regression model, *Figure* *2* *A* and *Table* *S1*) and Shannon's diversity (coefficient = 0.20, *P* < 0.05, linear regression model, *Figure* *2* *A* and *Table* *S1*). There was no difference in the evenness (Pielou's evenness) and phylogenetic diversity (Faith's PD). We observed no difference in any index for the alpha diversity across the SMI quartiles groups in females (*Figure* *2* *B* and *Table* *S1*).

To determine the difference between microbial communities in the quartile groups by SMI, we calculated the beta diversity using both phylogenic methods (UniFrac distance) and non‐phylogenetic (Bray–Curtis dissimilarity and Jaccard distance). In both sexes, there was no significant difference in all beta‐diversity indices across the SMI quartile groups (*Table* *S2*).

### Taxonomic profiling between Q1 and Q4 groups of skeletal muscle mass index

Collapsing ASVs at the phylum, class, order, family, genus and species level, we found the taxa with significantly high abundance in males with the highest quartiles of SMI (*Table* *3* and *Figure* *S2*). The relative abundance of Pasteurellales of order, including its family Pasteurellaceae, was more likely to be prevalent in males with the highest quartile (Q4) SMI values (*W* = 25, ANCOM‐II; coefficient = 1.908, *P* = 0.021, MaAsLin2). At the genus level, we found that *Haemophilus* (*W* = 146; coefficient = 1.909, *P* = 0.021) belonging to Pasteurellaceae and unclassified genus (*W* = 132; coefficient = 1.641, *P* = 0.031) belonging to Lachnospiraceae were more prevalent in males with the highest quartile SMI. We used the DADA2 tool to produce ASVs that could provide high‐resolution sample inference from Illumina amplicon data^20^ as well as the NCBI‐RefSeq database that was used for taxonomic assignment of the ASVs. The NCBI‐RefSeq provides the highest species‐level taxonomic entropy.^21^ Therefore, we could identify some species associated with SMI from this 16S rRNA sequencing data. At the species levels, the relative abundances of *Haemophilus parainfluenzae* (*W* = 258; coefficient = 1.910, *P* = 0.020), *Roseburia faecis* (*W* = 218; coefficient = 1.536, *P* = 0.032), *Eubacteriales* unclassified species (*W* = 289; coefficient = 2.541, *P* = 0.008) and *Lachnospiraceae* unclassified species (*W* = 240; coefficient = 1.627, *P* = 0.033) were more prevalent in the Q4 group than in the Q1 group, and the significances were robust in the results of ANCOM‐II (*W*) and MaAsLin2 (*P* < 0.05) (*Table* *3*). *Figure* *S2* shows the relative abundance of taxa the significantly associated with SMI in group comparisons in males.

**Table 3:** Taxa differed between the lowest quartile group (Q1) and the highest quartile group (Q4) for the skeletal muscle mass index in males.

Taxa levels	Taxonomic assignment^a^	ANCOM‐II	MaAsLin2	
		*W* ^b^	Coefficient^c^	*P* value
Phylum	—	—	—	—
Class	—	—	—	—
Order	**p_Proteobacteria;c_Gammaproteobacteria;o_Pasteurellales**	25	1.908	**2.1 × 10** ^**−2**^
Family	p_Firmicutes;c_Negativicutes;o_Acidaminococcales;f_Acidaminococcaceae	56	0.904	7.9 × 10^−1^
	**p_Firmicutes;c_Clostridia;o_Eubacteriales;_**	49	2.543	**8.1 × 10** ^**−3**^
	**p_Proteobacteria;c_Gammaproteobacteria;o_Pasteurellales;f_Pasteurellaceae**	41	1.908	**2.1 × 10** ^**−2**^
Genus	**p_Firmicutes;c_Clostridia;o_Eubacteriales;_;_**	157	2.546	**8.1 × 10** ^**−3**^
	**p_Proteobacteria;c_Gammaproteobacteria;o_Pasteurellales;f_Pasteurellaceae;g_*Haemophilus***	146	1.909	**2.1 × 10** ^**−2**^
	**p_Firmicutes;c_Clostridia;o_Eubacteriales;f_Lachnospiraceae;_**	132	1.641	**3.1 × 10** ^**−2**^
Species	**p_Firmicutes;c_Clostridia;o_Eubacteriales;_;_;_**	289	2.541	**8.3 × 10** ^**−3**^
	**p_Proteobacteria;c_Gammaproteobacteria;o_Pasteurellales;f_Pasteurellaceae;g_*Haemophilus*;s_*parainfluenzae***	258	1.910	**2.0 × 10** ^**−2**^
	**p_Firmicutes;c_Clostridia;o_Eubacteriales;f_Lachnospiraceae;_;_**	240	1.627	**3.3 × 10** ^**−2**^
	**p_Firmicutes;c_Clostridia;o_Eubacteriales;f_Lachnospiraceae;g_*Roseburia*;s_*faecis***	218	1.536	**3.2 × 10** ^**−2**^
	p_Firmicutes;c_Clostridia;o_Eubacteriales;f_Lachnospiraceae;g_*Blautia*;s_*faecis*	201	1.132	5.4 × 10^−1^

*Note*: Both ANCOM‐II and MaAsLin2 models were adjusted for age, BMI and regular physical activity. The Q4 group was set as the reference group and compared with the Q1 group. If the significance in the ANCOM‐II model is validated in the MaAsLin2 model, the taxa and *P* values are shown in bold.

Abbreviations: ANCOM, analysis of composition of microbiomes; BMI, body mass index; c_, class; f_, family; FDR, false discovery rate; g_, genus; MaAsLin2, multivariate association with linear models; NCBI‐RefSeq, National Center for Biotechnology Information (NCBI) Nucleotide and Taxonomy databases; o_, order; p_, phylum; s_, species.

^a^ NCBI‐RefSeq database was used for taxonomic assignment. No. of phylum: 13, no. of class: 26, no. of order: 35, no. of family: 67, no. of genera: 186 and no. of species: 325 in Q1 (*n* = 155) versus Q4 (*n* = 156) comparison analysis.

^b^ *W* = X for taxon k, and then *H*_0k_ is rejected X times. The *W* statistic for the significantly different taxa relative to more than 60% other taxa in each taxa level is represented (FDR *P* < 0.05, Benjamini–Hochberg method).

^c^ Exponentiation of the coefficients for the log‐transformed relative abundance of each taxon in the linear model adjusted for age, BMI and regular physical activity using MaAsLin2.

However, in females, we found no robust association between the SMI and specific gut microbiota on comparing the Q1 and Q4 groups. Although the relative abundance of the genera *Clostridium*, *Weissella* and *Lachnospira* and the species [*Eubacterium*] *eligens* were significantly different between the Q1 and Q4 groups in ANCOM‐II analysis, the significance was not validated in MaAsLin2 analysis (*Table* *4*).

**Table 4:** Taxa differed between the lowest quartile group (Q1) and the highest quartile group (Q4) for the skeletal muscle mass index in females.

Taxa levels	Taxonomic assignment^a^	ANCOM‐II	MaAsLin2	
		*W* ^b^	Coefficient^c^	*P* value
Phylum	—	—		—
Class	—	—	—	—
Order	—	—	—	—
Family	—	—	—	—
Genus	p_Firmicutes;c_Clostridia;o_Eubacteriales;f_Clostridiaceae;g_*Clostridium*	155	—	6.2 × 10^−1^
	p_Firmicutes;c_Bacilli;o_Lactobacillales;f_Lactobacillaceae;g_*Weissella*	147	0.830	5.0 × 10^−1^
	p_Firmicutes;c_Clostridia;o_Eubacteriales;f_Lachnospiraceae;g_*Lachnospira*	137	0.847	5.8 × 10^−2^
Species	p_Firmicutes;c_Clostridia;o_Eubacteriales;f_Clostridiaceae;g_*Clostridium*;_	275	1.762	8.7 × 10^−1^
	p_Firmicutes;c_Bacilli;o_Lactobacillales;f_Lactobacillaceae;g_*Weissella*;_	247	0.941	5.3 × 10^−1^
	p_Firmicutes;c_Clostridia;o_Eubacteriales;f_Lachnospiraceae;g_*Lachnospira*;s_[*Eubacterium*] *eligens*	227	0.859	5.7 × 10^−2^

*Note*: Both ANCOM‐II and MaAsLin2 models were adjusted for age, BMI and regular physical activity. The Q1 group was set as the reference group and compared with the Q4 group. If the significance in the ANCOM‐II model is validated in the MaAsLin2 model, the taxa and *P* values are shown in bold.

Abbreviations: ANCOM, analysis of composition of microbiomes; BMI, body mass index; c_, class; f_, family; FDR, false discovery rate; g_, genus; MaAsLin2, multivariate association with linear models; NCBI‐RefSeq, National Center for Biotechnology Information (NCBI) Nucleotide and Taxonomy databases; o_, order; p_, phylum; s_, species.

^a^ NCBI‐RefSeq database was used for taxonomic assignment. No. of phylum: 13, no. of class: 28, no. of order: 40, no. of family: 73, no. of genera: 197 and no. of species: 346 in Q1 (*n* = 107) versus Q4 (*n* = 108) comparison analysis.

^b^ *W* = X for taxon k, and then *H*_0k_ is rejected X times. The *W* statistic for the significantly different taxa relative to more than 60% other taxa in each taxa level is represented (FDR *P* < 0.05, Benjamini–Hochberg method).

^c^ Exponentiation of the coefficients for the log‐transformed relative abundance of each taxon in the linear model adjusted for age, BMI and regular physical activity using MaAsLin2.

### Inference of functions of gut microbiota between the Q1 and Q4 groups of skeletal muscle mass index

**Figure 3:**
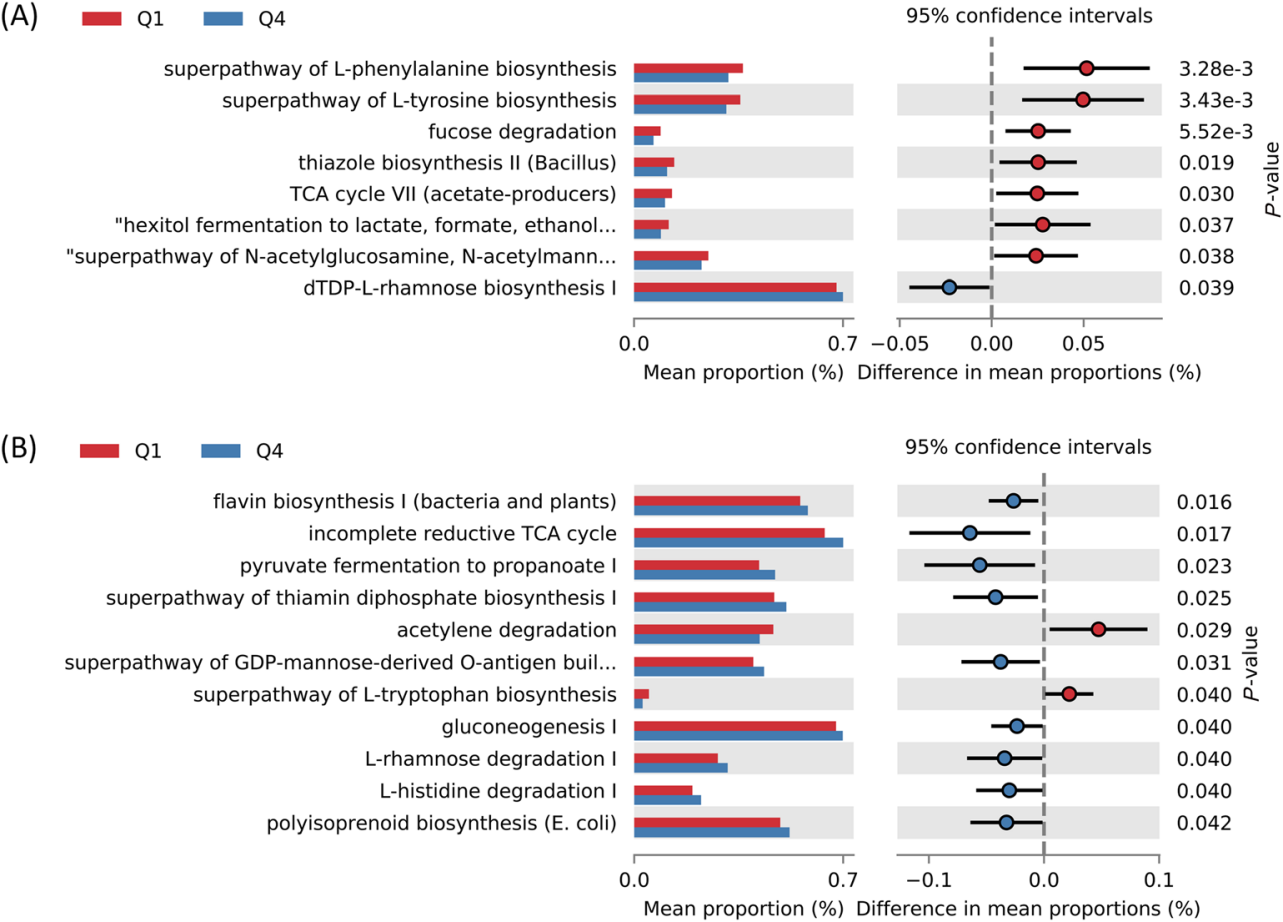
Difference in the predicted metabolic pathways between the Q1 and Q4 groups based on skeletal muscle mass index in (A) males and (B) females. Bar plots on the left side display the mean proportion of each pathway. Dot plots on the right show the differences in mean proportions between the two indicated groups based on *P* value. The *P* values have not been corrected with any multiple comparison method, and those with <0.05 are shown. GDP, guanosine‐5′‐diphosphate; TCA, tricarboxylic acid

To understand the gut microbial functions related to SMI, we used PICRUSt2 to infer putative metagenomes from the 16S rRNA gene profiles. After multiple comparison correction, we found no pathway that passed the significant threshold among 368 MetaCyc pathways (FDR *q* < 0.05). Instead, *Figure* *3* *A* shows the suggestive eight pathways with nominal significance in males (*P* < 0.05). Pathways related to amino acid biosynthesis (e.g., l‐phenylalanine and l‐tyrosine) and carbohydrate degradation (e.g., fucose degradation) were higher in the Q1 group than in the Q4 group in males (*P* < 0.01). Considering the *P* values with <0.05, pathways related to vitamin biosynthesis (e.g., thiazole) and generation of precursor metabolites and energy (e.g., tricarboxylic acid [TCA] cycle and hexitol fermentation to lactate, formate, ethanol and acetate) were higher in the Q1 group than in the Q4 group in males (*P* < 0.05), whereas carbohydrate biosynthesis (e.g., dTDP‐l‐rhamnose) was enriched in the Q4 group compared with that in the Q1 group in males (*P* < 0.05).

In females, we did not find any pathways with a nominal significance level of *P* < 0.01; however, we found 11 pathways with a nominal significance level of *P* < 0.05 (*Figure* *3* *B*). The pathways related to generation of energy (e.g., acetylene degradation) and amino acid biosynthesis (e.g., l‐tryptophan) were also higher in the Q1 group than in the Q4 group in females (*P* < 0.05), whereas pathways related to fermentation of pyruvate, vitamin biosynthesis (e.g., flavin and thiamin), carbohydrate biosynthesis (e.g., guanosine‐5′‐diphosphate [GDP]‐mannose and gluconeogenesis), carbohydrate degradation (e.g., l‐rhamnose) and amino acid degradation (e.g., l‐histidine) were enriched in the Q4 group compared with those in the Q1 group in females (*P* < 0.05).

## Discussion

This large population‐based study demonstrated that the gut microbiota was significantly associated with skeletal muscle mass in middle‐aged males. Although the presence of a gut–muscle axis has been recently hypothesized, there was limited evidence from only a few human studies in which the results should be interpreted with caution due to the lack of statistical power and a small sample size.^11^, ^12^ To the best of our knowledge, this is a first study to report sex‐specific differences in the diversity and composition of gut microbiota according to SMI in healthy population‐based study. Our results demonstrate that decreased gut microbial richness was independently associated with low SMI after adjustments for age, sex, BMI and regular physical activity, which was shown in males but not in females. Although in the present investigation, the differences in microbiota composition according to SMI were not significant in terms of beta diversity, we found that the highest SMI (Q4) group has the high abundance of beneficial bacterial taxa compared with the lowest SMI (Q1) in males.

At the species level, *H. parainfluenzae* and *R. faecis* were more abundant in the highest (Q4) group than in lowest (Q1) group in males. Previous studies reported that abundance of *H. parainfluenzae* is associated with the amelioration of insulin resistance and glucose tolerance status.^22^, ^23^, ^24^ Furthermore, a recent study by Chierico et al. presented that *H. parainfluenzae* was significantly associated with low fasting glucose and decreased insulin resistance.^22^ Increasing evidence supports that a high skeletal muscle mass is known to be inversely related to a risk of diabetes and insulin resistance.^25^, ^26^ Skeletal muscle is the primary organ of insulin‐mediated glucose disposal. Thus, high muscle mass is linked to increased anabolism and decreased catabolism. Conversely, declining muscle mass can negatively affect glucose metabolism with elevating insulin resistance. Therefore, the identification of enriched *H. parainfluenzae* might be a parameter for higher muscle mass with a better metabolic health.

We also found that *R. faecis* was significantly associated with the highest SMI (Q4) group, with showing a decreased abundance in the lowest SMI (Q1) group in males. This finding was in line with that in a previous report that *Roseburia* was relatively decreased in abundance among elderly sarcopenic subjects compared with that in healthy controls.^11^ *Roseburia* is one of the major microorganisms that produce an SCFA such as butyrate.^27^ Previous studies in both animal and human have linked *Roseburia* with anti‐inflammatory properties.^27^, ^28^ Butyrate has been reported to give a beneficial effect by anti‐inflammatory properties through down‐regulation of NF‐κB and STAT2 activity.^29^ Chronic low‐grade inflammation is one of the principal patho‐mechanisms for sarcopenia, characterized by decreased muscle mass.^30^ Low muscle mass has been known to be strongly linked to metabolic syndrome (MS).^31^ A recent study among patients with MS for analysing sex difference in gut microbiome demonstrated that a higher abundance of *Roseburia* was found in men than in women after 3 years of healthy diet intervention.^32^ Therefore, *Roseburia* might play a beneficial role in the metabolic effect of skeletal muscle with showing sex‐dependent feature. Our results show the association between *R. faecis* and SMI. Further studies are required to explore the causal relationship between gut microbiota and skeletal muscle mass.

The metabolic pathways of microbiota associated with SMI were inferred from the 16S rRNA gene sequencing data using PICRUSt2. The several metabolic pathways related to carbohydrate degradation, amino acid biosynthesis and vitamin biosynthesis were enriched in the lowest SMI group in males. In contrast, in females, the pathways related to amino acid biosynthesis were enriched in the lowest SMI group, but vitamin and carbohydrate biosynthesis was depleted in the lowest SMI group, although there was no overlap with the metabolic pathways found in males. The association between the functionalities of gut microbiota involved in amino acid metabolism and subjects with sarcopenia has been reported in human and animal model studies.^12^, ^33^ Siddharth et al. suggested that microbial‐derived dietary metabolic pathways including carbohydrate, lipid and vitamin metabolism could alter the metabolic status of the host and contribute to the physiological state in ageing and sarcopenia.^33^ They also reported that the gut microbiota of a pre‐sarcopenic model was distinct from those of the healthy and sarcopenic models, and pathways related to diet including metabolism of carbohydrate, protein, lipids and vitamin biosynthesis were associated with the pre‐sarcopenic samples. Previous studies reported that amino acid synthesis pathways were underrepresented in older individuals with a low muscle mass.^11^, ^12^ Decreased amino acids have been shown to be associated with a lower muscle mass in older individuals.^34^ We found that both sexes in the lowest SMI group had enriched biosynthesis of amino acids such as phenylalanine, tyrosine and tryptophan compared with the highest SMI group, which is consistent with findings from sarcopenic individuals.^12^ Previous studies have demonstrated that essential amino acids, such as phenylalanine and tryptophan, are necessary for the stimulation of muscle protein anabolism in the elderly.^35^ Synthesis and ingestion by the gut microbiota of these amino acids are essential for the maintenance of skeletal muscle because they are not produced naturally within the body. Therefore, physiology of skeletal muscle may be linked to a decline in amino acid synthetic pathway. In the view of host–microbiome symbiotic relationship,^36^ the gut microbiota of a middle‐aged host should actively produce amino acids for the synthesis of the host's skeletal muscle under harsh condition such as sarcopenia. With the same contexture, energy generation (e.g., TCA cycle and acetylene degradation) of both sexes was higher in the lowest SMI group than in the highest one, which might be necessary for the production of skeletal muscle of the host. Understanding the precise implications for human skeletal muscle metabolism and the role of the gut microbiota, however, will require further studies accounting for the human physiology, gut microbiome and the interactions at play.

Previous studies have reported the sex difference for skeletal musculature.^9^, ^19^ Therefore, we also conducted sex‐stratified analyses for all the analyses, the results of which demonstrated a positive association of the microbial alpha diversity and compositions with skeletal muscle mass only in males. Although the exact pathophysiology of sex difference in association between gut microbiota and muscle mass is not established yet, there are several possible explanations. In addition to the previous explanations, the sex differences of gut microbiome have been observed since birth. Chen et al. recently studied gut microbiota of twin infants, with longitudinal analysis, in which they observed that males and females were significantly dissimilar in diversity, compositions and metabolic profiles of gut microorganisms.^37^ From the beginning of lifetime, varieties of sex hormones, foot intakes, daily activities and many others can be related with sex differences of gut microorganisms.^38^ Especially, women had a drastic hormonal change during menopause in contrast to men, which induced a loss of skeletal muscle mass. The influence of gut microorganisms on skeletal muscle might be weak compared with the effects of sex hormones in women.

This study has several limitations. First, because this was a cross‐sectional study design, no exact cause–effect relationship could be confirmed. However, this study is the largest population‐based study to increase the generalizability of the findings. Second, this study included middle‐aged participants, which may have led to a selection bias. However, the age of the population showed a normal distribution in the bell curve. Moreover, we performed multivariate‐adjusted analysis including age as a possible confounding factor. Third, there were no data for muscle function or performance but only included muscle mass. Future studies must measure the grip strength and/or gait speed to determine sarcopenia. Fourth, we did not examine the effects of the fat mass, which can be a potential bias on the association between gut microbiota and SMI. A previous study among healthy Chinese children reported that relationship of gut microbiome between muscle parameters was mediated by body fat content.^10^ However, the cross‐sectional design of this study could not determine the temporal directionality of the association among fat mass, skeletal muscle mass and gut microbiota. Without validation of the temporal ordering of variables, the magnitude of the bias may be introduced by mediation analysis or conditioning on a collider, although we observed significant negative correlations between fat mass and SMI in both sexes. Further study is needed to elucidate the causal relationship between the variables in adult population. Fifth, the functional inference of our results should be carefully considered because we did not conduct the whole‐genome metagenomic or metatranscriptomics analyses and did not measure any metabolite. Metagenomic shotgun sequencing could expand our understanding of the species associated with SMI, their genes and functional potential. Nevertheless, this is the first population‐based study on sex difference with a large sample size conducted in adults. Our results demonstrate the association between the gut microbiota and the SMI, particularly in males, which reflects the differences in biodiversity and taxonomic composition across different SMIs. Future prospective studies must be conducted to explain the patho‐mechanisms and therapeutic implications. Lastly, muscle mass was measured by multi‐frequency BIA in this study. However, dual‐energy X‐ray absorptiometry (DEXA) is a well‐known technique for analysing body composition including muscle mass. Nevertheless, many recent studies showed that BIA was well correlated with the results of DEXA.^39^, ^40^ Moreover, the BIA has advantages of being easily applied for health screening method for a large population because of its rapid and relatively simple method with no need for radiation exposure and specialized radiologist.

In conclusion, the diversity and composition of the gut microbiota were positively associated with skeletal muscle mass in male participants, showing the highest muscle mass with increased alpha diversity and high relative abundance in taxa such as *H. parainfluenzae* and *R. faecis*. We suggest that controlling the gut microbiome is an important therapeutic target strategy for subjects, especially men with low muscle mass accompanied by functional decline or sarcopenia, to increase skeletal muscle mass and improve overall muscle function.

## Conflicts of interest

The authors declare no competing interests in relation to this study.

## Supporting information


**Figure S1.** Sequence read counts across the SMI quartile groups in (A) males and (B) females.
**Table S1.** Alpha diversity among groups based on the skeletal muscle mass index in males and females.
**Table S2.** Comparison beta diversity among SMI quartile groups based on SMI in males and females.
**Figure S2.** Relative abundance of the significantly associated taxa with SMI in males. (A) Order, (B) family, (C) genus, and (D) species levels.
