# Supplementary material for: Sex‐specific associations between gut microbiota and skeletal muscle mass in a population‐based study
Source: J Cachexia Sarcopenia Muscle. 2022 Oct 11;13(6):2908–19. doi: 10.1002/jcsm.13096 (PMC9745450; doi:10.1002/jcsm.13096)
Supplement: Supplementary file 1 — Figure S1. Sequence read counts across the SMI quartile groups in (A) males and (B) females. Table S1. Alpha diversity among groups based on the skeletal muscle mass index in males and females. Table S2. Comparison beta diversity among SMI quartile groups based on SMI in males and females. Figure S2. Relative abundance of the significantly associated taxa with SMI in males. (A) Order, (B) family, (C) genus, and (D) species levels. [file JCSM-13-2908-s001.pdf]

Supplementary Files

Sex-specific associations between gut microbiota and skeletal muscle mass in a population-based study

Journal name: Journal of Cachexia, Sarcopenia and Muscle

Chul-Hyun Park[1,2], Eun-Ju Lee[2], Hyung-Lae Kim[3], Yong-Taek Lee[1], Kyung Jae Yoon[1,2,4,5†], and Han-Na Kim[2,4,†]

Correspondence to:

Kyung Jae Yoon

Department of Physical and Rehabilitation Medicine, Kangbuk Samsung Hospital Sungkyunkwan University College of Medicine, Seoul, Republic of Korea

Medical Research Institute, Kangbuk Samsung Hospital, Sungkyunkwan University School of Medicine, Seoul, Republic of Korea

Department of Clinical Research Design and Evaluation, SAIHST, Sungkyunkwan University, Seoul, Republic of Korea

Biomedical Institute for Convergence at SKKU, Sungkyunkwan University School of Medicine, Suwon, Republic of Korea

Email: yoon.kjae@gmail.com; Tel: +82-2-2001-2284; Fax: +82-2-2001-1284

and

Han-Na Kim

Medical Research Institute, Kangbuk Samsung Hospital, Sungkyunkwan University School of Medicine, Seoul, Republic of Korea

Department of Clinical Research Design and Evaluation, SAIHST, Sungkyunkwan University, Seoul, Republic of Korea

Email: hanna147942@gmail.com; Tel.: +82-2-2001-1978

Supplementary methods

DNA extraction from fecal samples and 16S rRNA gene sequencing

Fecal samples were immediately frozen at –20 ℃ after collection and stored at –70 ℃ within 24 h. DNA extraction from fecal samples was performed within 1 month of storage using the MOBio PowerSoil® DNA Isolation Kit (MO BIO Laboratories, Carlsbad, CA, USA) using the bead beating step according to the manufacturer's instructions. Variable regions V3 and V4 of the 16S rRNA gene were amplified by polymerase chain reaction (PCR) with universal primers. Libraries were pooled for sequencing using the full complement of Nextera XT indices and paired-end sequenced on the Illumina MiSeq platform (Illumina, San Diego, CA, USA) according to the manufacturer's instructions [1].

The Divisive Amplicon Denoising Algorithm, version 2 (DADA2) plugin [2] of the Quantitative Insights into Microbial Ecology (QIIME2) package (version 2019.7, https://qiime2.org) [3], was used to perform sequence quality control and construct a feature table of amplicon sequence variants (ASVs) that are regarded as 100% operational taxonomic units (OTUs). We have applied the contingency-based filtering to remove features that are present in only a single sample in our feature table. Taxonomy was assigned to all ASVs by searching against the National Center for Biotechnology Information (NCBI) Nucleotide and Taxonomy databases (accessed on 9 June 2021) [4] using RESCRIPt [5] within QIIME2.

Statistical analysis

Previous data show that skeletal muscle mass differs according to sex [6]; therefore, we compared the SMI between males and females. The SMIs were compared using Student's *t*-tests. As the SMI differed by sex, association analyses between SMI and gut microbiota were conducted separately for males and females. To find out a linear trend of baseline characteristics according to increasing SMI quartiles, one-way analysis of variance (ANOVA) for continuous variables and χ^2 tests for categorical variables were performed. The right-skewed variables (insulin, glucose, and triglycerides) were log-

transformed, and a one-way ANOVA performed. We controlled for age, BMI, and regular physical activity to determine if there was a significant association between gut microbiota and skeletal muscle mass.

For diversity analysis, the feature table was rarefied to 2,019 sequences per sample by random subsampling in the QIIME2 package (version 2021.11, https://qiime2.org). To evaluate alpha diversity, we computed the number of ASVs observed in each sample, Faith's phylogenetic diversity (Faith's PD; measures of biodiversity that incorporate phylogenetic differences between species), Shannon's index (calculates richness and diversity using a natural logarithm) accounting for both evenness and richness, and Pielou's evenness (measures of relative evenness of species richness). The Estimated Marginal Means (EMMs) analysis was used to show the adjusted mean of each SMI quartile group controlling for covariates such as age, body mass index (BMI) and exercise. For the linear regression models, Q1 was set as the reference group and compared with the Q2, Q3, and Q4 groups, respectively. To estimate the dissimilarity between samples, β-diversity was calculated using the UniFrac distance to estimate dissimilarity among group members by incorporating the phylogenetic distances between ASVs. Unweighted and weighted UniFrac distances were calculated to determine the presence/absence and abundance of ASVs, respectively. Non-phylogenetic β-diversity indices such as Bray–Curtis dissimilarities were also used for abundance data. Pairwise permutational multivariate analysis of variance with 999 random permutations using *adonis* function was performed to test the significance of differences between groups. Age, BMI, and regular physical activity were included in the formular of *adonis* as covariates. Basic statistical analyses were performed using RStudio (version 1.3.1073, Boston, MA, USA), and plots of microbial diversity were depicted using the ggplot2 package (version 3.3.2) in RStudio.

To robustly investigate the significant differences in the relative taxa abundances from the phylum to species levels among groups, we used two statistical tools from R (version 4.0.2): analysis of composition of microbiomes (ANCOM)-II ((https://github.com/FrederickHuangLin/ANCOM, accessed on 17 May 2022) [7] and generalized linear models implemented in multivariate association with linear models (MaAsLin2) [8]. After adjusting for age, BMI and exercise, we compared the abundance of taxa between the Q1 and Q4 groups in a pairwise manner based on microbial diversity

results. ANCOM compares the relative abundance of taxa among groups by the log-ratio of the abundance of each taxon to that of the remaining taxa, one at a time. The final significance was expressed in the empirical distribution of W at each taxonomic level. We used the taxa-wise false discovery rate (FDR) option with the significance level set to FDR < 0.05 to generate W statistics and a threshold of 0.6 for declaring a significant association. For the MaAsLin2 models, Q1 was set as the reference group and compared with Q4. After adjusting for covariates such as age, BMI, and exercise, we estimated the exponentiated (exp) coefficients by comparing the highest quartile (Q4) of SMI to the lowest quartile (Q1).

To predict metagenome functional content from 16S rRNA gene surveys, we predicted the functional pathways from the MetaCyc metabolic pathway database [9] using PICRUSt2 (v2.4.2, accessed on 22 April 2020) [10]. The predicted functional pathways were compared among the groups using statistical analysis of taxonomic and functional profiles (STAMP) version 2.1.3. Statistical differences in the pathways were tested using Welch's t-test with a Benjamini–Hochberg FDR correction (q-value < 0.05) to adjust for multiple testing.

Supplementary Figure S1. Sequence read counts across the SMI quartile groups in (A) males and (B) females.

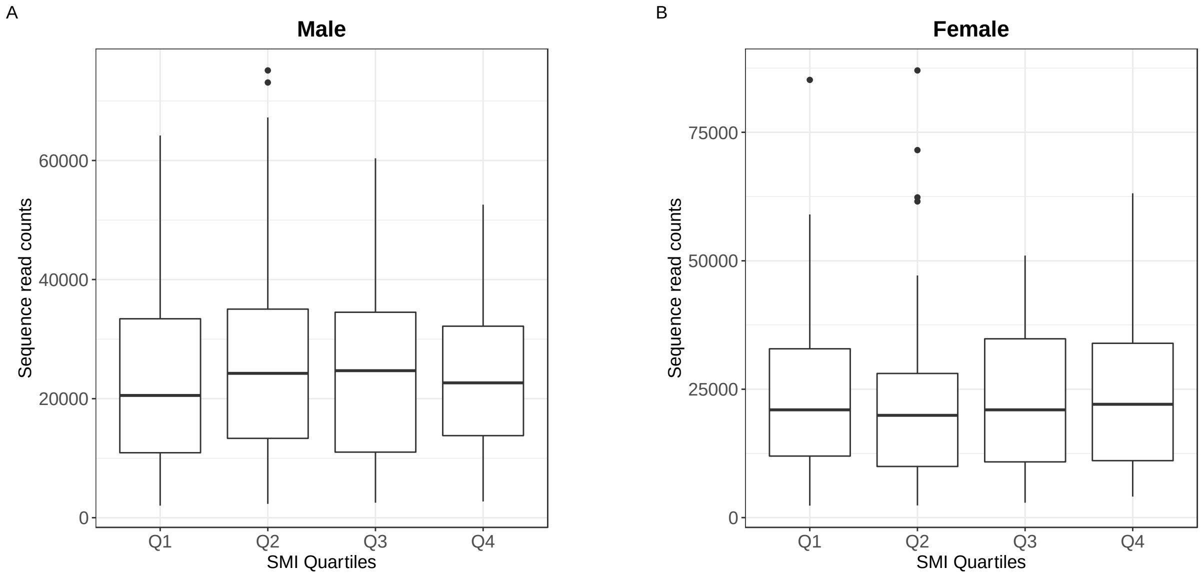

SMI, skeletal muscle mass index

Supplementary Table S1. Alpha diversity among groups based on the skeletal muscle mass index in males and females.

Sex	Alpha diversity indices		SMI quartile groups				Linear regression analysis	
			Q1	Q2	Q3	Q4	Adjusted R^2	*p*-value
Males (*n* = 621)	Overserved features	Mean ± SD	82.06 ± 41.62	88.50 ± 43.57	88.34 ± 39.07	89.72 ± 42.77		
		Coefficient (SE) [a]	1	6.70 (4.78)	8.21 (4.93)	**10.79 (5.40) ***	0.006	0.150
	Faith's PD	Mean ± SD	14.11 ± 3.39	13.68 ± 3.23	14.35 ± 3.02	14.34 ± 3.31		
		Coefficient (SE) [a]	1	0.60 (0.37)	0.76 (0.39)	0.81 (0.42)	0.007	0.131
	Shannon's diversity	Mean ± SD	4.95 ± 0.79	4.97 ± 0.81	5.09 ± 0.69	5.11 ± 0.70		
		Coefficient (SE) [a]	1	0.03 (0.09)	0.17 (0.09)	**0.20 (0.10) ***	0.013	0.035
	Pielou's evenness	Mean ± SD	0.81 ± 0.07	0.79 ± 0.08	0.81 ± 0.07	0.81 ± 0.06		
		Coefficient (SE) [a]	1	-0.01 (0.01)	-0.00 (0.01)	0.00 (0.01)	0.003	0.248
Females (*n* = 431)	Overserved features	Mean ± SD	92.55 ± 44.20	85.12 ± 42.08	94.67 ± 43.91	93.00 ± 42.75		
		Coefficient (SE) [a]	1	-6.98 (6.08)	3.53 (6.33)	3.45 (7.33)	0.016	0.058
	Faith's PD	Mean ± SD	14.74 ± 3.53	14.03 ± 3.28	14.96 ± 3.42	14.87 ± 3.40		
		Coefficient (SE) [a]	1	-0.66 (0.48)	0.35 (0.50)	0.37 (0.58)	0.021	0.026
	Shannon's diversity	Mean ± SD	5.01 ± 0.76	5.04 ± 0.79	5.18 ± 0.75	5.19 ± 0.65		
		Coefficient (SE) [a]	1	-0.06 (0.10)	0.10 (0.11)	0.15 (0.13)	0.014	0.076
	Pielou's evenness	Mean ± SD	0.81 ± 0.07	0.81 ± 0.07	0.81 ± 0.06	0.81 ± 0.06		
		Coefficient (SE) [a]	1	0.01 (0.01)	0.00 (0.01)	0.00 (0.01)	-0.012	0.969

[a] The coefficients were calculated in the linear regression analysis with adjusting for age, BMI, and regular physical activity as covariates.

* $p < 0.05$

SD, standard deviation; SE, ; BMI, body mass index; Faith's PD, Faith’s phylogenetic diversity

Supplementary Table S2. Comparison beta diversity among SMI quartile groups based on SMI in males and females.

Sex	Beta diversity indices	Df	SumsOfSqs	MeanSqs	F.Model	R^2	Pr(>F)
Males (*n* = 621)	Unweighted UniFrac distance	3	0.297	0.099	1.238	0.006	0.126
	Weighted UniFrac distance	3	0.371	0.124	0.712	0.003	0.698
	Jaccard distance	3	1.164	0.388	1.033	0.005	0.263
	Bray-Curtis Dissimilarity	3	1.196	0.399	1.076	0.005	0.203
Females (*n* = 431)	Unweighted UniFrac distance	3	0.272	0.091	0.944	0.007	0.553
	Weighted UniFrac distance	3	0.577	0.192	1.323	0.009	0.184
	Jaccard distance	3	1.073	0.358	0.956	0.007	0.784
	Bray-Curtis dissimilarity	3	1.168	0.389	1.067	0.007	0.228

Permutational multivariate analysis of variance (PERMANOVA) with the *adonis* function was performed with 999 permutations using each beta diversity index after adjusting for age, BMI, and regular physical activity as covariates.

BMI, body mass index; SMI, skeletal muscle mass index

Supplementary Figure S2. Relative abundance of the significantly associated taxa with SMI in males. (A) Order, (B) family, (C) genus, and (D) species levels.

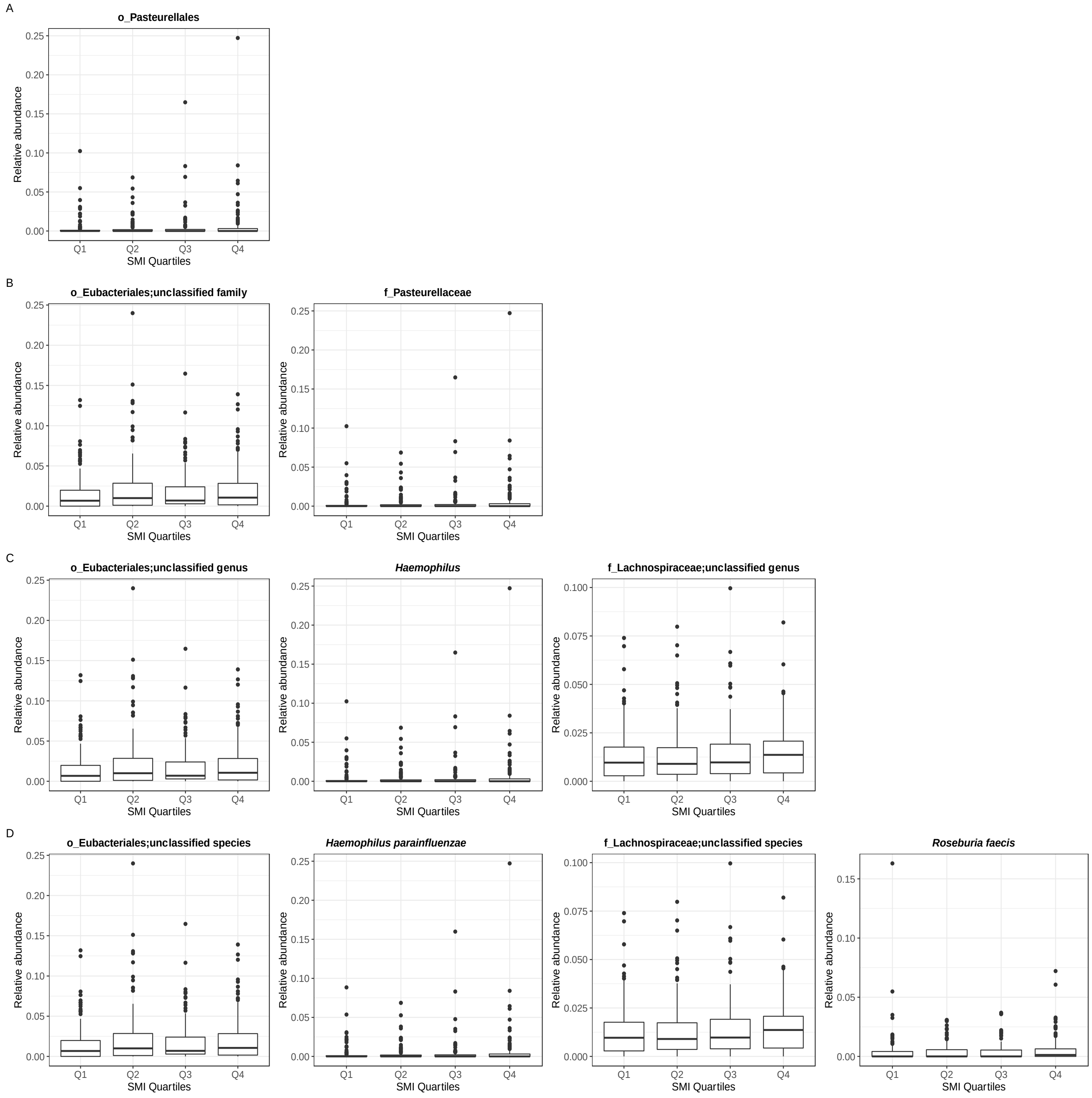

SMI, skeletal muscle mass index

Supplementary References

1. Kozich JJ, Westcott SL, Baxter NT, Highlander SK, Schloss PD. Development of a dual-index sequencing strategy and curation pipeline for analyzing amplicon sequence data on the MiSeq Illumina sequencing platform. Appl Environ Microbiol. 2013;79:5112-20. doi:10.1128/AEM.01043-13

2. Callahan BJ, McMurdie PJ, Rosen MJ, Han AW, Johnson AJ, Holmes SP. DADA2: High-resolution sample inference from Illumina amplicon data. Nat Methods. 2016;13:581-3. doi:10.1038/nmeth.3869

3. Bolyen E, Rideout J, Dillon M, Bokulich N, Abnet C, Al-Ghalith G, et al. Reproducible, interactive, scalable, and extensible microbiome data science using QIIME 2. Nat Biotechnol. 2019;37:852-7. doi:10.7287/peerj.preprints.27295v2

4. Coordinators NR. Database resources of the National Center for Biotechnology Information. Nucleic Acids Res. 2018;46:D8-D13. doi:10.1093/nar/gkx1095

5. Robeson MS, 2nd, O'Rourke DR, Kaehler BD, Ziemski M, Dillon MR, Foster JT, et al. RESCRIPt: Reproducible sequence taxonomy reference database management. PLoS Comput Biol. 2021;17:e1009581. doi:10.1371/journal.pcbi.1009581

6. Haizlip KM, Harrison BC, Leinwand LA. Sex-based differences in skeletal muscle kinetics and fiber-type composition. Physiology (Bethesda). 2015;30:30-9. doi:10.1152/physiol.00024.2014

7. Kaul A, Mandal S, Davidov O, Peddada SD. Analysis of Microbiome Data in the Presence of Excess Zeros. Front Microbiol. 2017;8:2114. doi:10.3389/fmicb.2017.02114

8. Mallick H, Rahnavard A, McIver LJ, Ma S, Zhang Y, Nguyen LH, et al. Multivariable association discovery in population-scale meta-omics studies. PLoS Comput Biol. 2021;17:e1009442. doi:10.1371/journal.pcbi.1009442

9. Baggerman MR, van Dijk DPJ, Winkens B, van Gassel RJJ, Bol ME, Schnabel RM, et al. Muscle wasting associated co-morbidities, rather than sarcopenia are risk factors for hospital mortality in critical illness. J Crit Care. 2020;56:31-6. doi:10.1016/j.jcrc.2019.11.016

10. Douglas GM, Maffei VJ, Zaneveld JR, Yurgel SN, Brown JR, Taylor CM, et al. PICRUSt2 for prediction of metagenome functions. Nat Biotechnol. 2020;38:685-8. doi:10.1038/s41587-020-0548-6
